# The effects of oral contraceptive usage on thrombin generation and activated protein C resistance in Saudi women, with a possible impact of the body mass index

**DOI:** 10.1371/journal.pone.0206376

**Published:** 2018-10-25

**Authors:** Abdulrahman B. O. Mohamed, Hilde Kelchtermans, Joke Konings, Jamilla van Daal, Anas Al Marzouki, Steve Harakeh, Bas de Laat

**Affiliations:** 1 Department of Pharmacology, King Abdul-Aziz University, Jeddah, Saudi Arabia; 2 Synapse Research Institute, Cardiovascular Research Institute Maastricht, Maastricht University, Maastricht, the Netherlands; 3 Department of Biochemistry, Cardiovascular Research Institute Maastricht, Maastricht University, Maastricht, the Netherlands; 4 Department of Obstetrics Gynecology, King Abdul-Aziz University, Jeddah, Saudi Arabia; 5 Special Infectious Agents Unit, King Fahd Medical Research Center, King Abdul-Aziz University, Jeddah, Saudi Arabia; Institut d'Investigacions Biomediques de Barcelona, SPAIN

## Abstract

**Background and objectives:**

The effect of oral contraceptive (OC) usage on coagulation has been studied worldwide. However, no such studies have been conducted in Saudi Arabia on Saudi women using OCs. The aim of this study was to investigate the effects of OC-induced changes of thrombin generation (TG) in the absence and presence of activated protein C (APC) or thrombomodulin (TM) in Saudi women.

**Methods:**

A total of 115 adult women, 47 on oral contraception (OC users) and 68 controls (not using OCs) were recruited from the obstetrics-gynecology outpatient clinic in Saudi Arabia. OCs that were used in this study include the following: Marvelon, Gynera, Cerrazetem, Yasmine, Microlut, Gracial and Diane. The plasma calibrated automated thrombinography (CAT) was used to determine TG which was expressed as endogenous thrombin potential (ETP; nM/min), lag time (min), peak (nM) and time-to-peak (ttpeak; min). In the presence of TM or APC, TG parameters were expressed relative to the parameters in the absence of TM or APC.

**Results and conclusion:**

As in other populations, our study demonstrated that OC usage induced prothrombotic changes in plasma of Saudi women, including resistance to the inhibitory actions of TM and APC. More specifically, OC usage in our population predominantly influenced TG and APC/TM sensitivity in overweight and obese individuals, a finding that needs confirmation in large cohort studies. The effects of APC and TM on TG parameters showed a positive association, and the correlation coefficients were higher in OC users for both ETP and peak values.

## Introduction

Due to some religious views that prohibit the use of birth control measures, oral contraceptive usage has been a controversial topic in Saudi Arabia and other communities around the world for a long time. Specifically, the recent change in socio-demographic landscape of the Saudi Arabian community, especially concerning women’s education and work, resulted in evolving needs of women in the country, with tendencies to birth spacing and consequently the use of oral contraceptives [1–5].

The combined estrogen and progestogen oral contraceptives (OCs) are known to increase the thromboembolic risk by unbalancing coagulation homeostasis and inducing a procoagulant state [6]. These effects were initially attributed to the estrogen dose [7, 8]. However, the type of progestogen used was also demonstrated to be a determinant of the thrombogenetic effect of oral contraception. Several studies comparing different classes and generations of progestogens concluded to have differential effects on coagulation factors and related thromboembolic risk [9–11]. Third-generation OCs containing progestogens such as desogestrel and gestodene are also associated with a 2 to 3 fold increase in thromboembolic risk [12–16].

The use of OCs has been associated with excessive thrombin generation (TG), as assessed by comparative studies of endogenous thrombin potential (ETP) between users and non-users, as well as between different progestogens [17]. This excess in TG is explained by increased levels of procoagulant factors such as factors VII, VIII and II with concomitant decrease in anticoagulant factors such as antithrombin and protein S [18, 19].

Another mechanism in OC-induced thrombotic tendency- especially with third-generation OCs- is the reduction in plasma sensitivity to activated protein C (APC) [17, 20–23]. Protein C is a precursor of the anticoagulant pathway, which is activated by the thrombin-thrombomodulin (TM) complex formation at the endothelial cell surface. After its activation and in concurrence with its cofactor protein S, APC inhibits procoagulant factors Va and VIIIa, thus down regulating TG [24–26]. The effect of OC use can be characterized by the acquired APC resistance, determined as a reduced anticoagulant action of APC in plasma of OC-users, which is more pronounced for third-generation OCs [11, 27].

The effect of OC usage on coagulation in the Saudi population has not been studied before. With the evolving needs of the Saudi women, we hypothesized the general characteristics of the population (such as age) and hence the effect of OC usage on coagulation, possibly differ from the previously studied populations. In this study, we therefore aimed to investigate the effects of OC-induced changes of TG in the absence and presence of APC or TM among Saudi women.

## Materials and methods

### Study population

The protocol used in this study was ethically approved by the institutional review board of King Abdul-Aziz University (KAU), Jeddah, and Kingdom of Saudi Arabia.

A total of 115 adult women (47 on oral contraception [OC users]) and 68 controls not using OCs) were recruited from the obstetrics-gynecology outpatient clinic at KAU Hospital, Jeddah. Individuals were eligible for inclusion if they were Saudi females. OCs that were used in this study include Marvelon, Gynera, Cerrazetem Yasmine, Microlut, Gracial and Diane. In both groups, participants on oral anticoagulant or anti-platelet drugs over the last month preceding the study as well as those who had a history of thrombosis or bleeding were excluded from this study. All participants gave full written informed consent according to the Helsinki declaration.

### Blood sample collection and preparation

Blood samples were collected by antecubital venipuncture into Vacutainer tubes containing 0.105 mol L−1 trisodium citrate (ratio 9:1; Becton Dickinson, Pont de Claix, France). Platelet-poor plasma (PPP) was obtained after double centrifugation of citrated blood (4000 × g for 10 min at 25°C) and kept frozen at −80°C until further analysis.

For the normal pooled plasma (NPP) blood from 10 healthy control donors was collected. After an initial centrifugation step (2500g, 5 min), plasmas were pooled, followed by ultra-centrifugation (10,000g, 10 min). Aliquots of 1 ml were snap-frozen in liquid nitrogen and stored at -80°C until analysis.

### Reagents

Z-Gly-Gly-Arg-aminomethylcoumarine (ZGGR-AMC) was purchased from Bachem (Basel, Switzerland). Recombinant tissue factor (TF) was from Innovin (Dade-Behring, Marburg, Germany). Synthetic phospholipids were from Avanti Polar Lipids Inc. (Alabaster, AL, USA). The calibrator, α2-macroglobulin-thrombin complex, was prepared as previously described [28]. Hepes buffers containing 5 mg/ml (BSA5) and 60 mg/ml (BSA60) bovine serum albumin were composed for dilution of the reagents or the substrate, respectively, as described by Hemker *et al*. [29]. Recombinant human TM and APC were a kind gift of Asahi Kasei Pharma (Japan) and Veronique Regnault (France), respectively.

### Calibrated automated thrombinography

The plasma calibrated automated thrombinography (CAT) was performed as described previously [28]. In short, 10 μl of the TF/phospholipid mixture was added to a well of a flat bottom 96-well polystyrene plate with either 10 μl of BSA5 buffer or TM or APC. The final concentrations were 1 pM TF, 4 μM phospholipids and 2.5 nM TM or APC. TM and APC concentrations were determined to be around the IC_50_ levels in NPP. In the calibrator wells, instead of reagents, 20 μl of calibrator (613 nM) was pipetted. In every well 80 μl of PPP was added. TG was started by the addition of 20 μl of FluCa (ZGGR-AMC at 2.5 nM and CaCl_2_ at 100 nM in BSA60 buffer) to the wells.

Fluorescent signals were measured at excitation/emission wavelengths of 390/460 nm. The data were acquired by the specialized software from Thrombinoscope (Maastricht, the Netherlands). Results of the TG test were expressed as endogenous thrombin potential (ETP; nM.min), lag time (min), peak (nM) and time-to-peak (ttpeak; min). ETP and peak data were normalized to the data obtained in NPP at 5 pM TF. In the presence of TM or APC, TG parameters were expressed relative to the parameters in the absence of TM or APC (lag time and ttpeak as ratio to corresponding parameter without APC or TM; peak and ETP as % inhibition by TM or APC).

### Statistical methods

Statistical analysis was performed using the Statistical Package for Social Sciences version 24.0 for Windows (SPSS Inc., Chicago, IL, USA). One way ANOVA or t test was carried out to analyze the difference of baseline parameters, % inhibition by TM and % inhibition by APC with BMI numerical values (Kg/m2) in total population as well as separately in OC users and controls. Normal distribution of the data was confirmed by Kolmogorov normality test. Participants were categorized into two groups due to the small sample size: group 1 included normal and underweight (BMI <25); and group 2 included overweight (25≥BMI<30) and obese (≥30). To assess the effect of BMI on TG, t- test was used to compare TG parameters before and after the addition of TM or APC between the two BMI categories, in OC users and controls. Results of the two previous analyses are presented as mean ± standard deviation (SD).

## Results

### Characteristics of the study population

Of the total of 115 Saudi females, 47 (41.59%) were using OCs and general characteristics are shown in Table 1. Age distribution showed no significant difference between the two groups (p = 0.368). The two groups were comparable regarding BMI (p = 0.273) and blood group (O group versus non-O group; p = 1.000). Among the women on OCs, the majority (51.1%) were on 3^rd^ generation of OCs while 19.1% were not able to remember the name of their OC. The mean ± SD duration of OC treatment was 3.04±3.99 years.

**Table 1:** Characteristics of the study population.

Parameter	Value	OC users		Controls	
		Number	%	Number	%
Age	< 30	16	34.00	20	29.40
	30 – <40	20	42.60	25	36.80
	40 –<45	10	21.30	16	23.50
	≥50	1	2.10	7	10.30
Age	Mean, SD	35.53	6.54	37.68	1.00
BMI (kg/m^2^)	<25 (Normal/ underweight)	20	42.55	13	19.12
	≥25 (Obese/ overweight)	25	53.19	27	39.70
	Unknown	2	4.26	28	41.18
OC generation	2^nd^	7	14.90	-	
	3^rd^	24	51.10	-	
	4^th^	7	14.90	-	
	Unknown	9	19.10	-	
Duration of OC (years)	Mean, SD [range=3 months-17 years]	3.04	3.99	-	

%: percentage; BMI: body mass index; SD: standard deviation; OC: oral contraceptive

### Effect of OC use on TG parameters

Comparison of TG parameters between the two study groups showed a higher thrombotic risk profile among OC users, characterized by a shorter lag time (p<0.001) and ttpeak (p = 0.004), and an increased ETP (p = 0.032) and peak (p = 0.043) compared to females not using OCs, (Table 2).

**Table 2:** Thrombin generation parameters in OC users versus controls.

Parameter	OC users		Controls		p-value
	Mean	SD	Mean	SD	
Lag time (min)	3.44	0.94	4.05	0.82	0.001*
ETP (%)	131.8	29.8	125.8	33.2	0.032*
Peak (%)	242.0	100.1	218.3	85.7	0.043*
ttPeak (min)	6.95	1.98	7.98	1.72	0.004*

SD: standard deviation; ETP: Endogenous Thrombin Potential; OC: oral contraceptives; ttpeak: time to peak.

* Statistically significant result (p<0.05)

### Effect of OCs on the sensitivity to APC/TM as measured by TG

In the presence of TM or APC, a reduction in ETP was observed in both study groups. Significant differences in TG parameters between the two groups included a higher ETP_TM_ (p = 0.004) and ETP_APC_ (p = 0.001) as well as peak_TM_ (p = 0.007) and peak_APC_ (p = 0.001) and a reduced lag time_TM_ (p = 0.021*) and lag time_APC_ (p<0.005*) in women using OCs as compared to those who are not. The inhibition of the TG parameters peak and ETP by TM and APC was significantly higher in females without OCs compared to females using OCs (Table 3).

**Table 3:** The effect of TM or APC on thrombin generation parameters in women using OC versus controls.

Parameter	OC users		Controls		p-value
	Mean	SD	Mean	SD	
Lag time_TM_ (min)	3.4	0.8	3.7	0.8	0.021*
*Ratio*^1^	0.98	0.07	0.93	0.08	0.003*
Lag time_APC_ (min)	3.9	1.1	4.6	1.2	0.008*
Ratio*	1.16	0.15	1.15	0.08	0.830
ETP_TM_	77.1	47.9	56.2	27.7	0.004*
*Inhibition (%)*^1^	43.9	25.7	55.9	17.3	0.004*
ETP_APC_	80.3	47.5	54.3	31.2	0.001*
*Inhibition (%)*^1^	42.8	24.7	58.5	20.0	0.000*
Peak_TM_	166.4	113.8	116.0	80.3	0.007*
*Inhibition (%)*^1^	36.8	21.6	50.8	16.3	0.000*
Peak_APC_	152.0	117.4	90.4	75.8	0.001*
*Inhibition (%)*^1^	45.5	23.5	63.2	18.2	0.000*

SD: standard deviation; ETP: Endogenous Thrombin Potential; TM: thrombomodulin; APC: activated protein C

^1^ In the presence of TM or APC, TG parameters were expressed relative to the parameters in the absence of TM or APC (lag time as ratio to corresponding parameter without APC or TM; peak and ETP as % inhibition by TM or APC).

* Statistically significant result (p<0.05)

Looking at all included individuals regardless of their OC usage, the effects of TM and APC (% inhibition) showed moderate positive relationship for lag time (r = 0.464; p<0.0001), and strong positive relationships for ETP (r = 0.872; p<0.0001) and peak (r = 0.840; p<0.0001). In a separate analysis, the correlation between % inhibition by TM and APC was higher in OC users compared to the controls, for both ETP (r = 0.931 versus 0.786) and peak (r = 0.899 versus 0.727), respectively (Table 3).

### Effect of BMI on TG

The analysis of data showed no effect of BMI on TG in the control population, neither before nor after the addition of TM or APC (Table 4). However in the case of OC users, a higher BMI was associated with a significant increase in both ETP (p = 0.003) and peak (p = 0.006) After adding TM or APC, the percentage inhibition by TM/APC of both the peak and ETP was significantly reduced in the obese group compared to the non-obese group, illustrating an increased APC resistance in obese OC users compared to non-obese users. (Table 4).

**Table 4:** Effect of BMI on thrombin generation in OC users and controls.

Parameter	Group		Weight category (BMI)						p-value
			Normal/underweight			Obese/overweight			
			Mean	SD		Mean	SD		
**Baseline**		**N**			**N**			**N**	
Lag time	OC Users	**44**	3.6	0.6	**19**	3.2	1.1	**25**	0.172
	Control	**40**	3.9	0.8	**13**	3.9	0.9	**27**	0.840
ETP	OC Users	**44**	117.9	26.5	**20**	143.9	28.6	**24**	0.003*
	Control	**40**	117.1	12.3	**13**	129.6	39.2	**27**	0.273
Peak	OC Users	**45**	198.9	67.0	**20**	280.7	110.8	**25**	0.006*
	Control	**40**	197.6	50.3	**13**	229.9	92.1	**27**	0.162
**TM**									
Lag time	OC Users	**42**	3.5	0.5	**17**	3.2	0.9	**25**	0.123
	Control	**40**	3.7	0.7	**13**	3.7	0.9	**27**	0.890
ETP (%I)	OC Users	**42**	53.9	23.2	**18**	34.6	25.0	**24**	0.015*
	Control	**40**	52.8	15.0	**13**	52.5	18.3	**27**	0.955
Peak (%I)	OC Users	**43**	44.2	19.3	**18**	29.9	21.6	**25**	0.031*
	Control	**40**	48.6	11.6	**13**	46.4	19.1	**27**	0.706
**APC**									
Lag time	Users	**42**	4.4	0.9	**17**	3.6	1.2	**25**	0.019*
	Control	**40**	4.5	1.0	**13**	4.5	1.4	**27**	0.955
ETP (%I)	Users	**42**	54.2	20.6	**18**	33.3	24.7	**24**	0.006*
	Control	**40**	52.4	21.4	**13**	52.1	20.1	**27**	0.966
Peak (%I)	Users	**42**	56.3	18.2	**18**	36.4	24.3	**24**	0.006*
	Control	**40**	56.8	19.3	**13**	56.9	18.9	**27**	0.971

%I: Percentage inhibition

* statistically significant result (p<0.05)

## Discussion

The effect of OC usage on coagulation has been studied extensively worldwide in the past. However, this is the first study that investigated the effect of OC on thrombin generation in Saudi women.

Clinically, the use of OCs has been demonstrated to induce venous thrombosis as well as arterial thrombosis [6, 30], and is considered as the leading cause of venous thrombosis among young women [31]. In published studies comparing the different TG parameters between OC users and controls, small but significant differences were noted, including increased ETP and peak values and shortened lag time and ttpeak values in females using OCs [26, 32].

Additionally, inhibitory actions of TM and APC proved to be significantly reduced in females using OCs as compared to the control population. Previously, an increased APC sensitivity ratio (APC_sr_) was reported in women using OCs versus men and women not using OCs [33]. Another study demonstrated a weaker APC-induced inhibition of TG in women using OCs of the 3^rd^ generation as compared to those not using OCs [11]. The authors referred to residual ETP in presence of APC, which was found to be almost 3 times higher in women using OCs versus controls (28.0% versus 9.5%, respectively) and comparable to that observed in heterozygous APC-resistant subjects (29.0%) [27]. Our results showed higher levels of residual ETP among OC users, which may be related to the difference in APC and TM concentrations used, as well as in population characteristics and genetic predisposition.

Several data indicate that resistance to APC is a significant risk factor of venous thrombosis. Both men and women with thromboembolic events (cases) were shown to display higher APC resistance profiles in comparison to the controls [34]. Interestingly, the APC resistance remained significantly higher in cases versus controls even after exclusion of subjects with factor V-Leiden in the male population. However, in the female population after exclusion of factor V-Leiden carriers, APC resistance was also high in the controls. This observation was explained by the higher proportion of OC users among women in the control group, compared to the patients [35]. These relative increases in APC resistance profile among OC users without a history of thromboembolic events is consistent with our findings, and suggest that OC users have an increased risk for thromboembolic events. Similar to our findings, another study found an increase of baseline ETP in women using OCs by comparison to men and women not using OCs [33], along with a significantly shortened lag time and ttpeak and increase in peak height [31].

In both OC users and controls the level of resistance to APC was correlated to the resistance to TM, although this correlation was more evident in OC-users. Given the similarities in results upon addition of either TM or APC, the mechanism involved in TM resistance are hypothesized to be similar to the ones that are involved in APC resistance. Based on literature data, the reduced levels of protein S may underline this correlation. Indeed, one study demonstrated that norgestimate- and gestodene-containing OCs induce a significant decrease in circulating TM, in addition to a decrease in protein S activity and increase in circulating factor VIIa [36]. This suggests that the observed APC resistance upon addition of either APC or TM may be explained, at least partially, by an OC-induced decrease of protein S levels.

In our study, comparative analysis between the three OC generations (2^nd^ versus 3^rd^ versus 4^th^) showed no statistically significant difference in any of the studied parameters of TG, before or after the addition of either TM or APC (results not shown). However, our study was not designed to determine differences between OC generations and hence sample size is too small to draw firm conclusions. Previous research found no differences in baseline ETP between users of different OC formulations, but demonstrated that drospirenone-containing 3^rd^ generation OCs are likely to have more inhibitory action on APC than 2^nd^ generation OCs [33]. Similarly, another study demonstrated that 3^rd^ generation OCs (desogestrel-containing) have a greater APC resistance profile and induced more impairment to the anticoagulant pathway in comparison to 2^nd^ generation OCs (levonorgestrel-containing) [34].

Our study provides indications that OC use has a supplemental prothrombotic effect in combination with obesity. The findings suggest that overweight and obesity are associated with higher baseline TG profile including greater peak height and ETP, as compared with women with normal weight. This suggests that TG regulation by TM and APC is likely to be impaired in obese women; while it is relatively conserved in non-obese ones. High BMI was associated with venous thromboembolism [13]; and the risk of developing thromboembolic events is 3-fold greater for a BMI>25kg/m2 and more than 5-fold greater for a BMI>30kg/m2 [26]. Comparably to our study, a study by Rosing et al. investigated the effect of both BMI and body fat percentage on TG and found positive relationship with TG parameters including peak and ETP [27]; however, these relationships were only observed in female participants and not in males [27]. Similarly, a study found significant association of body fat percentage with thrombotic risk in female but not in males [26].

Important to note is that our study was originally not designed to study the impact of BMI on the observed procoagulant effect of OC usage on coagulation and hence the power calculations were not performed for this sub-analysis. We therefore acknowledge that the sample size is insufficient to draw firm conclusions in this direction. However, given the possible clinical implications, we think it is important to report these preliminary findings to encourage other research groups to confirm our findings in large (existing) cohorts. Additionally, we feel it may be interesting to verify a possible effect of the type or generation of OC on the observed BMI impact, as this may support decisions about the choice of the type/generation of OC in women with high BMI.

A further limitation of our study is the wide variation in duration of OC usage among study participants (3 months up to 17 years). Although interesting to investigate, the limited sample size impedes extracting meaningful information regarding the effect of the duration on the thrombogenic effect of OC usage. To carefully predict the risk of venous or arterial thrombosis linked to OC usage, additional studies investigating the impact of the duration of OC usage by sensitive techniques as thrombin generation are warranted.

## Conclusions

Our study demonstrates that OC usage induces prothrombotic changes in plasma of Saudi women, including resistance to the inhibitory actions of TM and APC.

The effects of APC and TM on TG parameters positively correlated, and correlation coefficients were higher in OC-users for both ETP and peak values. This suggests that TM and APC resistance mechanisms are interrelated and involve the same factors. Interestingly, OC usage in our population predominantly influences TG and APC/TM sensitivity in obese individuals. Given the small sample size in our study, this finding needs to be confirmed in large (existing) cohorts.
